# Pulmonary Embolism Presenting as Recurrent Syncope and Visual Disturbances in a Patient With Protein C Deficiency: An Atypical Emergency Department Presentation

**DOI:** 10.7759/cureus.99743

**Published:** 2025-12-20

**Authors:** Tarun Rana, Nidhi Kaeley, Parina Tejpal, Sonu Yadav, Deepanjan Mudi

**Affiliations:** 1 Department of Emergency Medicine, All India Institute of Medical Sciences, Rishikesh, Rishikesh, IND; 2 Department of Pulmonary Medicine, All India Institute of Medical Sciences, Rishikesh, Rishikesh, IND

**Keywords:** acute pulmonary embolism, emergency medicine, inherited thrombophilia, point-of-care-ultrasound, syncope, visual disturbances

## Abstract

Pulmonary embolism (PE) is a potentially life-threatening cardiovascular emergency that can present with atypical, non-respiratory manifestations, leading to diagnostic delay. We describe a 41-year-old previously healthy man who presented with recurrent episodes of syncope, giddiness, and transient blurring of vision over five days. Neurological and ocular examinations, as well as non-contrast computed tomography (CT) of the head, were unremarkable. Point-of-care echocardiography revealed global ventricular hypokinesia, dilated right atrium and ventricle, and reduced tricuspid annular plane systolic excursion (TAPSE=12 mm). CT pulmonary angiography confirmed acute bilateral PE. Further work-up demonstrated decreased protein C activity (40%), consistent with hereditary protein C deficiency. The patient was treated promptly with anticoagulation and achieved full recovery. This case highlights the protean nature of PE, which may masquerade as a neurological illness in the absence of classical respiratory or hemodynamic signs. Bedside echocardiography served as a critical diagnostic adjunct, allowing early recognition of right ventricular dysfunction and facilitating timely intervention. The detection of an inherited thrombophilia underscores the importance of evaluating for hypercoagulable states in young patients presenting with unprovoked venous thromboembolism. Emergency physicians should therefore maintain a high index of suspicion for PE in patients with unexplained syncope or transient neurological symptoms. Early imaging and bedside echocardiography remain pivotal for rapid diagnosis, and thrombophilia screening aids in guiding secondary prevention strategies.

## Introduction

Pulmonary embolism (PE) is a life-threatening cardiovascular emergency and the third most common cause of acute cardiovascular death, following myocardial infarction and stroke [1]. Despite advancements in imaging, PE remains frequently underdiagnosed due to its non-specific presentation. The classic triad of dyspnea, pleuritic chest pain, and tachycardia occurs in fewer than 20% of patients, leading to significant diagnostic delays [2].

Syncope is a key atypical manifestation, reported in 10%-20% of acute PE cases [3]. It often reflects a large thrombus burden causing transient reduction in cardiac output. The Pulmonary Embolism in Syncope Italian Trial (PESIT) trial highlighted this link, identifying PE in nearly 17% of patients hospitalised for a first episode of syncope [4]. Rare neurological manifestations, such as seizures or visual disturbances, can further obscure the diagnosis by mimicking stroke or seizure disorders [5].

In young patients or those with unprovoked thrombosis, the aetiology may stem from inherited thrombophilias. Protein C deficiency is a rare but significant genetic risk factor for venous thromboembolism (VTE). Protein C is a vitamin K-dependent anticoagulant that regulates the clotting cascade by inactivating Factors Va and VIIIa. A deficiency in this protein leads to a hyper-coagulable state, predisposing individuals to recurrent deep vein thrombosis (DVT) and pulmonary embolism [6].

The diagnostic challenge is compounded when patients present with neurological or cardiac-mimicking symptoms, often resulting in extensive negative work-ups before PE is considered. In this landscape, point-of-care ultrasound (POCUS) has become an essential bedside tool to identify right ventricular dysfunction and expedite the diagnosis [7].

We report the case of a young man with recurrent syncope and visual disturbances, ultimately diagnosed with bilateral PE secondary to protein C deficiency. This case underscores the necessity of considering PE and underlying thrombophilia in the differential diagnosis of unexplained syncope.

## Case presentation

A 41-year-old man, non-smoker with no known comorbidities, presented to the ED with two episodes of loss of consciousness over five days, associated with recurrent giddiness and blurring of vision. He denied chest pain, palpitations, dyspnoea, cough, headache or seizures.

**Figure 1:**
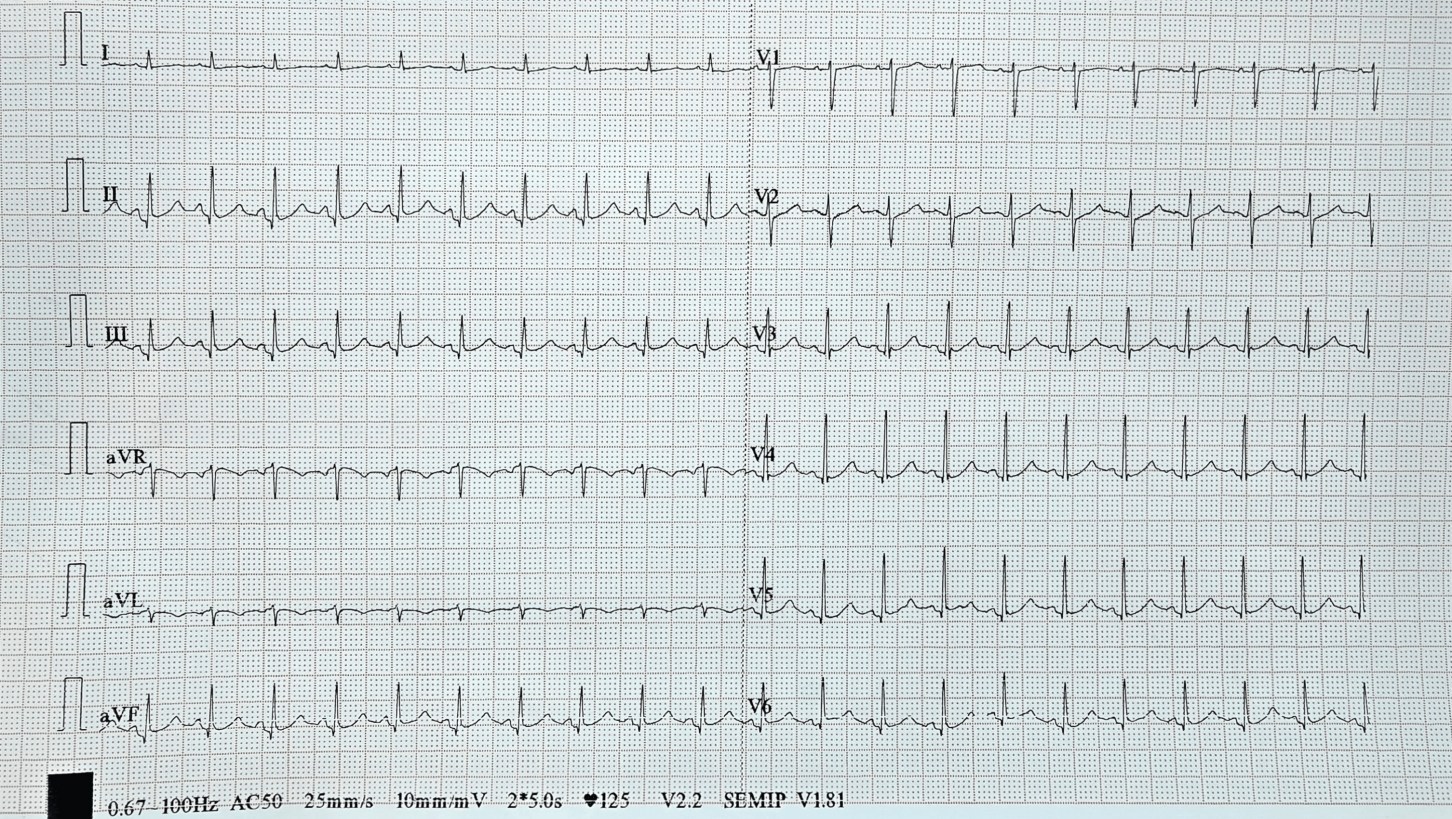
Electrocardiograph showing sinus tachycardia with a heart rate of 120/min

On arrival, he was tachycardic but normotensive and mildly hypoxemic on room air, but otherwise vitally stable (Table 1). Neurological examination, including head impulse-nystagmus-test of skew (HINTS) test, was normal. Systemic examination of the respiratory, cardiovascular system and the per abdominal examination was nonsignificant (Table 2). Non-contrast computed tomography (NCCT) head and ocular evaluation were unremarkable. ECG showed sinus tachycardia without ischemic changes (Figure 1). Syncope score scales were nonsignificant (Table 3).

**Table 1:** Patient's vitals on arrival

Vitals (on arrival)	
Blood pressure	130/70 mmHg
Pulse rate	120/min
Oxygen saturation	99% at room air
Respiratory rate	18/min
Glasgow Coma Scale	15/15

**Table 2:** Systemic examination HINTS: Head impulse-nystagmus-test of skew.

Neurological examination	Conscious and oriented to Time, place and person. Bilateral pupil - 3 mm, equally reactive to light. HINTS examination - Negative; no focal neurological deficit
Respiratory examination	Trachea central in position; bilateral lung fields- normal vesicular breath sounds; no added breath sounds
Cardiovascular examination	Normal s1, s2 heard; no added heart sounds; no murmurs
Abdominal examination	Soft, non-tender abdomen with normal bowel sounds

**Table 3:** Syncope scales EGSYS: Evaluation of Guidelines in Syncope Study.

San Francisco syncope rule score	0
EGSYS score	0 ( non cardiac cause likely)

**Figure 2:**
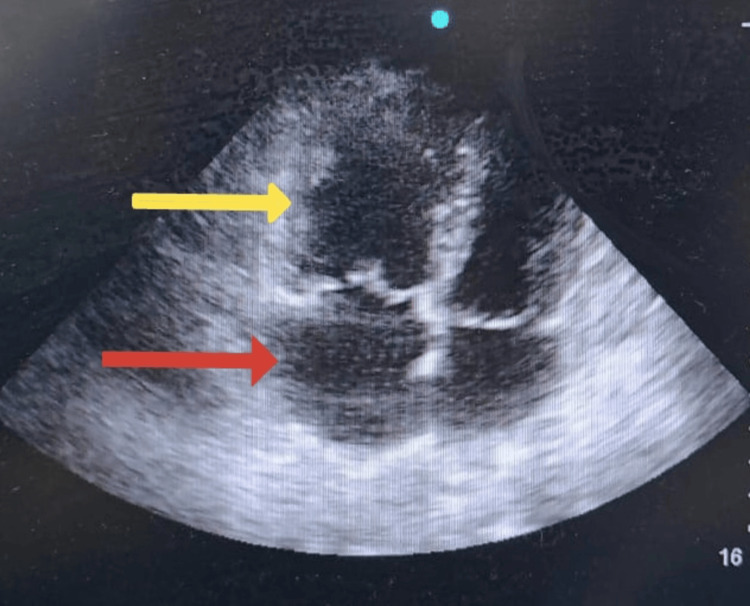
2D echocardiogram showing dilated right atrium (red arrow) and right ventricle (yellow arrow).

Bedside point-of-care echocardiography demonstrated a left ventricular ejection fraction of 45% with global hypokinesia, severe pulmonary hypertension, right atrial and ventricular dilatation, mild tricuspid regurgitation, and a tricuspid annular plane systolic excursion (TAPSE) of 12 mm (normal≥17 mm), indicating right ventricular dysfunction (Figure 2). High-sensitivity troponin I was elevated at 750 ng/l (normal <34 ng/l) and D-dimer was 1980 ng/ml (normal <500 ng/ml). The patient's coagulation profile showed decreased protein C activity at 40% (70%-140%), with normal prothrombin time (PT), activated partial thromboplastin time (aPTT), international normalised ratio (INR), and fibrinogen values within the normal range. Deep venous thrombosis (DVT) scan was negative.

**Figure 3:**
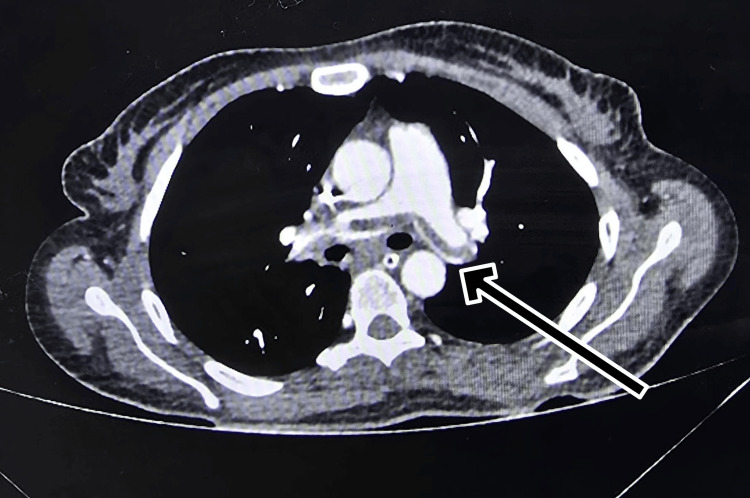
CT pulmonary angiography of the chest showing acute pulmonary embolism involving the bilateral main pulmonary arteries.

The CT pulmonary angiography confirmed acute pulmonary embolism in the bilateral main pulmonary arteries with extension into multiple segmental branches (Figure 3).

He was initiated on unfractionated heparin, a bolus of 80 units/kg given followed by intravenous infusion of 18 units/kg/hour with close hemodynamic monitoring and adjusted to maintain aPTT of 1.5-2.5 times the control (normal: 60-80 seconds). Given the presence of right ventricular dysfunction and elevated cardiac troponin but preserved systemic blood pressure, the case was classified as intermediate- to high-risk PE. In view of the patient’s stable hemodynamic status, systemic thrombolysis was deferred, and conservative management with anticoagulation was pursued.

Supportive therapy included oxygen supplementation, analgesia, and close monitoring for signs of decompensation. Over the subsequent days, the patient demonstrated progressive clinical and echocardiographic improvement, allowing transition to oral anticoagulation with warfarin. Regular follow-up ensured stable INR levels and progressive recovery.

Given the identification of protein C deficiency as the underlying thrombophilic state, a plan for extended anticoagulation was made to prevent recurrence. At three months, the patient was asymptomatic, with improved exercise tolerance and normalisation of right ventricular size and pulmonary pressures on follow-up echocardiography.

## Discussion

PE is known as "the great masquerader" due to its diverse clinical presentations that frequently mimic neurological or cardiac disorders. While the classic triad of symptoms is well known, it is absent in the majority of patients [2]. Syncope is a particularly challenging atypical presentation, occurring in up to 20% of acute PE cases [3]. As highlighted by the PESIT trial, PE is a frequent cause of hospitalisation for first-episode syncope, though the universal applicability of these findings remains a subject of debate [4].

In our case, the patient’s clinical picture was dominated by recurrent syncope and visual disturbances, with a notable absence of respiratory complaints. Syncope in PE typically signifies a hemodynamically significant event, resulting from a sudden reduction in cardiac output due to right ventricular (RV) obstruction. Similarly, visual symptoms, though rare, are thought to arise from transient global cerebral hypoperfusion or, in cases of patent foramen ovale, paradoxical embolism [5]. The predominance of these neurological symptoms initially obscured the diagnosis, leading to a negative neuroimaging work-up.

This diagnostic deadlock highlights the critical role of POCUS in the emergency department. While ECG and physical examination may be non-specific, bedside echocardiography can rapidly identify signs of RV strain - such as dilatation or McConnell’s sign - prompting confirmatory imaging with computed tomography pulmonary angiography (CTPA). In this patient, the identification of RV strain and elevated troponin levels indicated intermediate- to high-risk PE, as troponin elevation correlates with myocardial injury and adverse prognosis [7].

A pivotal aspect of this case was the identification of the underlying etiology. In young patients presenting with unprovoked venous thromboembolism (VTE), screening for inherited thrombophilia is essential. Protein C deficiency is a significant risk factor identified in this patient. Protein C is a vitamin K-dependent glycoprotein synthesised by the liver that, when activated, degrades Factors Va and VIIIa, thereby inhibiting the coagulation cascade. A deficiency in protein C, whether congenital or acquired, disrupts this regulatory mechanism, leading to a prothrombotic state [6]. Identifying this deficiency is vital, as it dictates the need for extended or lifelong anticoagulation to prevent recurrent life-threatening events. Several case reports in the literature have highlighted protein C deficiency as a rare but important cause of unprovoked PE, especially in young or otherwise healthy individuals. Alhenc-Gelas et al. reported a 24-year-old man with massive PE and hereditary protein C deficiency who recovered with anticoagulation therapy [8]. A case from India by Maqbool et al. illustrated PE along with myocardial infarction in a 37-year-old man with protein C deficiency, emphasising the value of screening for inherited thrombophilia in unexplained venous thromboembolism [9]. Collectively, these reports reinforce that protein C deficiency, though uncommon, should be considered in young patients with unprovoked or recurrent PE. More recently, cases continue to be reported underlining the clinical relevance of inherited PC deficiency as a cause of unprovoked PE. For instance, Zhang et al. described a patient with a novel PROtein C (PROC) gene intronic mutation who developed PE, confirmed on CT angiography and protein C assay, and recovered with anticoagulation [10]. Fatema and colleagues reported a young individual with recurrent PE plus deep vein thrombosis in the absence of traditional risk factors [11]. Similarly, Jauhar et al. highlighted that PC deficiency may present as multi-system thromboses sometimes obscuring classical PE presentation [12]. These reports underscore that hereditary PC deficiency remains a relevant and often under-recognised risk factor for PE, particularly in young or otherwise low-risk individuals, substantiating the rationale for thrombophilia screening in such scenarios.

Management in this case adhered to current European Society of Cardiology (ESC) guidelines, which recommend immediate anticoagulation for confirmed PE, reserving systemic thrombolysis for patients with hemodynamic instability [1]. The patient’s successful recovery underscores the importance of a broad differential diagnosis. Clinicians must maintain a high index of suspicion for PE in patients with unexplained syncope or neurological symptoms and should consider underlying thrombophilias like protein C deficiency in young, otherwise healthy individuals.

## Conclusions

PE is a "great masquerader" that can present atypically with syncope and visual disturbances, easily mimicking primary neurological disorders. This case illustrates that the absence of classic respiratory symptoms should not preclude the diagnosis of PE. Emergency physicians must maintain a high index of suspicion, particularly when the initial neurological work-up is unrevealing. Bedside echocardiography serves as an invaluable tool in this context, capable of identifying right ventricular dysfunction and accelerating definitive diagnosis. Furthermore, this case highlights the necessity of investigating underlying etiologies; in young patients with unprovoked thrombosis, screening for hypercoagulable states - such as protein C deficiency - is vital for guiding long-term anticoagulation and preventing life-threatening recurrence.
